# Mental health clinicians’ perceptions on patient motivations and intervention engagement for prenatal cannabis use: A mixed methods study

**DOI:** 10.1016/j.dadr.2025.100334

**Published:** 2025-04-19

**Authors:** Maha N. Mian, Monique B. Does, Andrea Altschuler, Andrea Green, Deborah R. Ansley, Carley Castellanos, Asma H. Asyyed, Derek D. Satre, Kelly C. Young-Wolff

**Affiliations:** a Suffolk University, Boston, MA, United States; b Department of Psychiatry and Behavioral Sciences, Weill Institute for Neurosciences, University of California, San Francisco, CA, United States; c Kaiser Permanente Northern California, Division of Research, Pleasanton, CA, United States; d Sacramento Medical Center, Kaiser Permanente Northern California, Sacramento, CA, United States; e Regional Offices, Kaiser Permanente Northern California, Oakland, CA, United States

**Keywords:** Cannabis, Marijuana, Perinatal, Pregnancy, Intervention, Substance use treatment, Harm reduction, Motivational interviewing, Mental health

## Abstract

**Background:**

Mental health clinicians are uniquely suited to support and provide important insights about substance use among pregnant patients. This mixed-methods study explored how mental health clinicians perceive and address prenatal cannabis use.

**Methods:**

Participants were licensed mental health clinicians from Kaiser Permanente Northern California’s Early Start perinatal substance use screening and counseling program. Participants aimed to support patients’ goals for a healthy pregnancy. ESS completed a survey (*N* = 26; 100 % Female; 73.1 % White; *M*_age_=48.1) and semi-structured interview (*n* = 14) on their perceptions about patients’ prenatal cannabis use and engagement in the ES program. Interviews were recorded, transcribed coded, and thematically analyzed.

**Results:**

Survey results indicated clinicians perceive that nausea/morning sickness was the most common motive for prenatal cannabis use, and pregnant individuals were most likely to get information about prenatal cannabis use from their peers. Survey and interview results indicated clinicians most often used motivational interviewing, harm reduction, and psychoeducation to address cannabis use. Clinicians reported on psychotherapeutic factors (patient readiness, therapeutic rapport, and mental health support) that facilitate engagement and willingness to quit and/or reduce cannabis use during pregnancy. Other themes included ESS utilization of expertise in complementary mental health topics to support their work.

**Conclusions:**

In this mixed-methods study, clinicians described several approaches to increase pregnant patients’ willingness to engage in perinatal substance use interventions, including eliciting motives for cannabis use and using patient-centered interventions focused on establishing rapport and addressing readiness to quit. Future interventions for patients might incorporate harm reduction and psychoeducation, address motivations for use and readiness to engage in care, emphasize peer support, and support the implementation of complementary interventions.

## Highlights

• Mental health clinicians report on prenatal cannabis use intervention engagement.
• Clinicians use MI, harm reduction, and psychoeducation for prenatal cannabis use.
• Patient readiness, rapport, and mental health support are facilitators.
• Clinicians utilize complementary mental health expertise to support their work.

## 1 Introduction

Cannabis is one of the most used substances during pregnancy, and use rates are rising (Brown et al., 2023, Young-Wolff et al., 2019). Prenatal cannabis use is associated with adverse neonatal outcomes, including low offspring birth weight, and may contribute to neurodevelopmental consequences (Metz et al., 2017, Paul et al., 2021, Sharapova et al., 2018). Some individuals may maintain cannabis use throughout pregnancy, and clinicians find addressing prenatal cannabis use to be uniquely challenging (Panday et al., 2022). Many pregnant individuals who use cannabis report that it alleviates pregnancy-related symptoms—including pain, poor sleep, loss of appetite, and nausea—and is a more desirable therapeutic option than using prescription medications or behavior change (Chang et al., 2019). Some individuals remain ambivalent about abstaining from cannabis during pregnancy and believe that there are little to no harms associated with use (Foti et al., 2023, Macario and Thomas, 2022).

Importantly, pregnant people want to have a safe and healthy pregnancy. Focus groups on prenatal cannabis use found that pregnant women want to have open conversations with their clinicians about cannabis use and are looking for reliable information about specific risks and effects of prenatal use (Foti et al., 2023, Jarlenski et al., 2016). Survey work has also examined attitudes about prenatal cannabis use among clinicians (Kansagara et al., 2020, Szaflarski et al., 2020), with results indicating that clinicians report discomfort discussing aspects of cannabis use with patients; furthermore, qualitative studies on physicians’ and nurses’ perspectives find that counseling around prenatal cannabis use is not universal and conversations may be more likely to emphasize legal concerns than health risks (Holland et al., 2016, Holland et al., 2016, Panday et al., 2022). Little work has explored or utilized mixed-method approaches to understand and evaluate the perspectives of mental health clinicians and the psychotherapeutic factors involved in addressing prenatal cannabis use.

Mental health clinicians are uniquely positioned to provide critical information about pregnant patients’ motivations for cannabis use and willingness to participate in interventions addressing substance use and/or mental health. At Kaiser Permanente Northern California (KPNC), the Early Start (ES) perinatal substance use screening and intervention program is embedded within obstetrics clinics as part of standard, comprehensive prenatal care. The mission of ES is to support pregnant individuals in having a safe and healthy pregnancy, based on a patient’s particular needs and goals, which may range from abstinence to harm reduction for those who are not willing to quit (e.g., strategies intended to mitigate harms associated with use). ES specialists (ESS) are licensed mental health clinicians who offer information and resources to support perinatal health. ES begins with a comprehensive intake process to understand a patient’s goals regarding substance use during pregnancy, and can provide interventions, including psychoeducation and harm reduction, which may be particularly helpful for those needing support to reduce their use. This well-established, integrated intervention program is associated with improved health outcomes for both mother and newborn (Goler et al., 2008, Goler et al., 2012). ESS can identify critical points for intervention and have essential insights on factors that are associated with successful patient engagement and navigating conversations about prenatal cannabis use. Importantly, understanding the perspectives of clinicians can provide a critical opportunity to examine how experiences of patients may overlap, and even more consequentially, differ from their clinicians. These insights may guide clinicians to further develop areas that patients and clinicians both find impactful, and they may also address where clinicians may adjust their approach or introduce new strategies. A comprehensive approach that can capture general trends in clinical approaches for prenatal cannabis use as well as deeply explore the nuanced strategies clinicians use can provide useful and practical clinical insights.

The present study addressed this key gap in the literature by exploring the perspectives and experiences of mental health clinicians who work with individuals who use cannabis during pregnancy in a state where adult cannabis use is legal. We aimed to survey clinicians about their perspectives on why pregnant individuals use cannabis, and to interview a subset of these clinicians to learn more about identifying the factors that lead to greater patient engagement in intervention.

## 2 Methods

### 2.1 Study setting

The study took place at KPNC, a large integrated healthcare system serving a diverse 4.6 million diverse patient population, representative of the northern California region (Davis et al., 2023, Gordon, 2020, Kaiser Permanente, 2023). Demographically, 23.7 % identify as Hispanic or Latino, 44 % as Non-Hispanic White, 22.1 % as Non-Hispanic Asian, 6.8 as Non-Hispanic Black, 2.2 % as Non-Hispanic Multiracial, 0.8 % as Non-Hispanic Pacific Islander, 0.4 % as Non-Hispanic American Indian or Alaskan Native.). KPNC encompasses 11 service areas, which include urban, suburban, and rural communities. Both recreational and medical cannabis use are legal in California. California penal code (California Penal Code § 11165.13, 2023, California Penal Code § 11166, 2023) indicates that a positive toxicology test on its own is not sufficient for healthcare providers are required to make a report to the Department of Social Services, only if a positive toxicology test for the presence of cannabis is accompanied with other risk factors.

Eligible participants were ESS, licensed mental health professionals, employed during study recruitment (03/2023–10/2023). ES is a substance use screening and intervention program established in the 1990s, embedded within obstetrics clinics as part of standard prenatal care at KPNC, and includes a screening questionnaire and urine toxicology test. Pregnant patients who screen in are referred to ES for an initial assessment. Interventions are brief, and time-limited to the course of the perinatal period. Based on the assessment, ESS provide recommendations for follow-up care and collaborate with patients to determine the course of care depending on goals during pregnancy.

### 2.2 Participants and study design

The study team contacted all ESS employed in KPNC during the study time window (N = 45) through email and invited potential participants to complete a confidential online survey. Participants who completed the survey were asked if they were also willing to participate in a follow-up interview. The purpose of the survey was to capture trends in ES approaches, with the interview component designed to explore nuances of clinical decision-making and the specific strategies clinicians employed. Interested participants were contacted by the research team to schedule a 60-minute interview hosted on Microsoft Teams, a HIPAA compliant teleconference platform. Interviews were conducted by study author MM and followed a semi-structured interview guide, developed by MM, MBD, AA, and KYW. Materials were reviewed by ES collaborators (AG, DRA) prior to implementation. The semi-structured format allowed participants to respond to planned questions and raise new topics for discussion. Participants received a $45 electronic gift card for completion of the survey and an additional $45 for completion of the interview. Interviews were recorded, de-identified, and professionally transcribed before coding. Participants provided informed consent before completing survey and interview. All procedures were in accordance with and approved by the KPNC Institutional Review Board and followed the Consolidated criteria for Reporting Qualitative research (COREQ) guidance for qualitative research (Tong et al., 2007).

### 2.3 Survey measures

Survey measures were adapted from prior literature, indicated below, and modified by MM and KYW for the current study.

#### 2.3.1 Demographic characteristics and training

Participants provided demographic information, including age, race/ethnicity, sex, gender, education, clinical experience, and years of ESS experience.

#### 2.3.2 Perceptions of patient motivations and knowledge sources

Participants reported on patients’ motivations for prenatal cannabis use (adapted from Daniels et al., 2022) and where they perceived patients sourced information about cannabis (adapted from Ziemianski et al., 2015) and benefits of cannabis use (adapted from Westfall et al., 2006). Participants reported on any endorsement of items (check all the apply) as well as the single most endorsed item (select one).

#### 2.3.3 Interventions

Participants reported on: a) commonly provided cannabis interventions and b) other interventions for pregnancy-related concerns (sleep, pain, mood, nausea). For each intervention, participants rated their willingness to recommend the intervention to patients using a four-point Likert scale, where higher scores indicated greater willingness. Participants reported on any endorsement of items (check all that apply) and the most important item (select one). Participants indicated which intervention models they used most often and which factors in intervention selection were most helpful. Response options for interventions were developed from prior qualitative work with focus groups of pregnant patients who engaged in prenatal cannabis use (Foti et al., 2023), as well as input from our clinician collaborators in the ES program. Interventions are listed in Table 3, and include motivational interviewing, harm reduction, and psychoeducation, among others.

### 2.4 Interview guide

The interview asked about participants’ training, how they initiate conversations about prenatal cannabis use with patients, develop intervention goals, and barriers/facilitators to intervention engagement.

### 2.5 Data analysis

Demographic information for race/ethnicity and degree was consolidated to preserve the confidentiality of participants (Table 1). Quantitative analyses were descriptive and included frequencies, averages, and ranges. Qualitative analyses included thematic coding to identify themes of interviews (Braun and Clarke, 2006, Nowell et al., 2017). The research team met regularly to discuss preliminary themes and determine thematic saturation (14 interviews). Researchers developed a codebook through an iterative process to develop themes/subthemes from findings from qualitative work with ES patients, field notes, preliminary themes, and an initial review of transcripts. Team members independently reviewed and coded transcripts and then met to achieve consensus on the codebook and coding (5 transcripts). Once consensus was reached, team members independently and manually coded the remaining 9 transcripts using a thematic analysis approach (MM, MD, AA, KYW each coded 2–3) with NVivo Qualitative Analysis Software (Version 14).

**Table 1:** Early Start Specialist (ESS) demographic characteristics and clinical experience.

		Survey Sample (n = 26)	Interview Sample (n = 14)
		N (%) or M (SD) [range]	
Age		48.1 (8.02) [35,65]	47.3 (9.27) [35,65]
Race/Ethnicity	African-American/Black or Asian-American/Asian or Multiracial	7 (26.9 %)	5 (35.7 %)
	White/ Caucasian	19 (73.1 %)	9 (64.3 %)
Gender	Female	26 (100 %)	14 (100 %)
Education	Master’s Degree	21 (80.8 %)	12 (85.7 %)
	Doctoral Degree	4 (15.4 %)	1 (7.1 %)
Degree	Marriage and Family Therapist or MS in Psychology	7 (26.9 %)	5 (35.7 %)
	Licensed Clinical Social Worker	14 (53.0 %)	7 (50.0 %)
	PsyD or PhD	5 (19.2 %)	2 (14.2 %)
Years of clinical experience		21.5 (7.6) [8,44]	21.3 (10.0) [8,44]
Years as Early Start Specialist		9.1 (6.4) [1,23]	8.3 (6.2) [2,21]

## 3 Results

### 3.1 Sample

Forty-five ESS were contacted, and 26 participated in the survey (*N* = 26; 100 %Female; 73.1 %White; *M*_age_=48.1; Table 1). About half the sample were licensed social workers (53 %). On average, ESS had 21.5 (*SD*=7.6, *range*=8, 44) years of clinical experience and 9.1 (*SD*=6.4, *range*=1, 23) years of experience in the ES program. We conducted interviews with a subset of 14 participants, after which thematic saturation was reached. Characteristics of those who completed the interviews were similar to the overall sample (Table 1).

### 3.2 Survey results

#### 3.2.1 Patient perceptions of prenatal cannabis use

ESS reported patients are motivated to use cannabis as a substitute for pharmaceuticals (100 %), and to relieve morning sickness/nausea (100 %), improve appetite (100 %), and reduce anxiety (100 %; Table 2). When asked to identify the most common patient motive, ESS selected relieving morning sickness (69.2 %). ESS reported patients obtain cannabis information from multiple sources, and perceived peers as the most common source of information (53.8 %).

**Table 2:** ESS perceptions of patient motivations for prenatal cannabis use and sources of information.

	Any endorsement	Endorsed as most common motive
Motives for use	N (%)	N (%)
Substitution for pharmaceuticals	26 (100 %)	1 (3.8 %)
Substitution for alcohol/nicotine/other drugs	12 (46.2 %)	0 (0 %)
Relieve morning sickness/hyperemesis gravidarum/nausea	26 (100 %)	18 (69.2 %)
Improve appetite	26 (100 %)	2 (7.7 %)
Treat aches or pain	24 (92.3 %)	0 (0 %)
Relieve depression	18 (69.2 %)	0 (0 %)
Treat sleep disturbances/insomnia	24 (92.3 %)	1 (3.8 %)
Reduce anxiety	26 (100 %)	2 (7.7 %)
For recreational or relaxation	22 (84.6 %)	2 (7.7 %)
Other: Habit/routine/pressure from significant other	1 (3.8 %)	0 (0 %)
Other: Relieve stress, take care of kids, relieve irritability, connect with partner	1 (3.8 %)	0 (0 %)
Perceived patient sources for getting information about cannabis		
Peers	24 (96.3 %)	14 (53.8 %)
Family	24 (92.3 %)	2 (7.7 %)
Budtenders/Dispensary/Cannabis Club	19 (73.1 %)	0 (0 %)
Clinicians	14 (53.%)	0 (0 %)
Online forums	18 (69.2 %)	4 (15.4 %)
Social Media	18 (69.2 %)	2 (7.7 %)
Online learning program	2 (7.7 %)	0 (0 %)
Online resources	12 (46.2 %)	4 (15.4 %)
Workshops	0 (0 %)	0 (0 %)
Pamphlet or materials from clinician	7 (26.9 %)	0 (0 %)
Peer-reviewed literature	2 (7.7 %)	0 (0 %)
Other (own research/ “research”)	4 (15.4 %)	0 (0 %)

#### 3.2.2 ESS interventions

ESS reported using several interventions to address prenatal cannabis use, including psychoeducation (100 %) and motivational interviewing (MI;100 %), which were endorsed most often (Table 3). Psychoeducation (M=4, SD=0, range=4,4) and Other (M=4, SD=0, range=4,4) had the highest ratings for ESS’ willingness to implement, followed by MI (M=3.96, SD=0.2, range=3, 4) and cognitive behavioral therapy (M=3.94, SD=0.24, range=3,4). ESS identified patients’ readiness/motivation to change as the most frequently endorsed factor for choosing an intervention to address prenatal cannabis use (100 %; Table 4).

**Table 3:** ESS interventions to address prenatal cannabis use.

	Interventions typically used	Willingness to recommend/implement intervention
	**Any endorsement**	**Scale range: 1 (very unlikely) = 4 (very likely)**
Interventions for prenatal cannabis use	**N (%)**	**M (SD) [range]**
Psychoeducation	26 (100 %)	4 (0) [4,4]
Motivational Interviewing	26 (100 %)	3.96 (.2) [3,4]
Contingency Management	8 (30.8 %)	3.62 (.52) [3,4]
Cognitive Behavioral Therapy	17 (65.4 %)	3.94 (.24) [3,4]
Psychotherapy	18 (69.2 %)	3.5 (.62) [2,4]
Alcoholics Anonymous/12 step	14 (53.8 %)	3.29 (.61) [2,4]
AMRS (Addiction Medicine Recovery Services)	22 (84.6 %)	3.5 (.74) [2,4]
Other clinic referral (including Psychiatry)	20 (76.9 %)	3.5 (.69) [2,4]
Other⁎	3 (11.5 %)	4.0 (0) [4,4]
Interventions as alternative to prenatal cannabis use for pregnancy symptoms		
	N (%)	M (SD) [range]
Psychoeducation	23 (88.5 %)	3.7 (.7) [1,4]
Cognitive Behavioral Therapy	10 (38.5 %)	3.7 (.48) [3,4]
Specialized/Targeted Psychotherapeutic Interventions (e.g., Cognitive Behavioral Therapy (CBT)-Insomnia, CBT for Chronic Pain)	10 (38.5 %)	3.6 (.52) [3,4]
Other Psychotherapy	13 (50 %)	3.54 (.52) [3,4]
Other clinic referral	18 (69.2 %)	3.5 (.62) [2,4]
Other⁎⁎	8 (30.8 %)	3.86 (.38) [3,4]

⁎ Other = “Stress/Harm reduction”; “harm reduction”

⁎⁎ Other=  Referral or consult with MD or OB (4 responses), pregnancy support group (1 response)

**Table 4:** Factors for considering intervention.

	For prenatal cannabis use (any endorsement)	For pregnancy related symptoms (any endorsement)
	**N (%)**	**N (%)**
Patient burden (if offered outside of KP)	12 (46.2 %)	13 (50 %)
Patient's readiness or motivation to change	26 (100 %)	2 (7.7 %)
Time/duration to deliver	13 (50 %)	16 (61.5 %)
Format	11 (42.3 %)	12(46.2 %)
Delivery method	13 (50 %)	12 (46.2 %)
Amount of content	8 (30.8 %)	9 (34.6 %)
Content is abstinence-focused	13 (50 %)	11 (42.3 %)
Content is harm-reduction focused	20 (76.9 %)	4 (15.4 %)
Cost	6 (23.1 %)	12 (46.2 %)
Other⁎	2 (7.7 %)	0 (0)

⁎ **Other** = “Judgment free”; “underlying reason for use”

ESS similarly rated interventions that address pregnancy-related symptoms in lieu of using cannabis (Table 3). ESS reported using psychoeducation (88.5 %) and other clinic referrals (69.2 %) most often. Other interventions, which included “referral or consult with medical doctor or obstetrics,” were rated highest for willingness to implement (M=3.86, SD=0.38, range=3,4). ESS rated time/duration of intervention as the most frequently considered factor for choosing an intervention to address pregnancy-related symptoms (61.5 %).

### 3.3 Qualitative results

We identified four major themes based on thematic analysis of interview transcripts that contextualize ESS’ approach to patients’ cannabis use; themes are discussed below (Table 5).

**Table 5:** Qualitative themes.

Theme	Illustrative quotes
Theme 1: Utilizing the initial clinical encounter	*“I had one patient I worked with who was smoking cannabis and also vaping pretty regularly. And they were like, ‘You know, the cannabis just does so much for me. I’m having a hard time imagining letting that go. But with vaping, I feel like I’m ready. In my mind, it’s riskier to baby. I feel more committed to that. I’ve been able to quit that before.’ So, for that patient, it was very obvious: ‘Okay, let’s help you get the vaping down.’ She couldn’t do both at once; that felt overwhelming. So really, the biggest thing is probably meeting them where they’re at and getting some traction based on what they can do.”*
Theme 2: Relying on patient-centered approaches to address prenatal cannabis use	“*So there are different rationales behind their [patients’] use and I try to understand that*—*because they often have either really good intent or desperate intent to survive, and if it’s coming from more managing difficult mental health symptoms, I really try to frame it as their survival skill, and we often use like, ‘You’re self-medicating,’ but it’s really their survival tool; and continue they may not be ready to give up on this survival tool that has worked for them because learning other forms of coping skills. So we talk about these things and really use motivational interviewing to see if it’s something they want, to actually to use to achieve the same outcome, or maybe we can explore different ways to achieve the same outcome without really having this sense of doubt or sense of shame about their cannabis use.”*
	“*When I get patients who say that they’re using cannabis for those pregnancy symptoms like nausea and that type of thing, we talk about how it could actually be contributing to their nausea. And so we start working on a plan to rule that out, because sometimes people just have a really crappy pregnancy and it has nothing to do with cannabis. But they won’t know until they quit. And most of them, given that information and that autonomy to be able to decide if they want to do that, will try it. And they’ll see if it makes a difference or not.”*
Theme 3: Leveraging expertise in complementary areas	*[On the value of trauma-informed training]: “Being able to recognize it [trauma] would be really helpful because that helps identify the root causes of people’s use, right? If we could target that, then we don’t even really have to talk much about the actual substance, but let’s talk about the root cause of why you’re using. I became EMDR-trained and I feel like that transformed how I work with patients in Early Start.”*
	*“Just showing them ways that they can manage without cannabis…my big thing is I always ask them to at least try. And when we meet again, they report out… I always tell them what to expect, something like, ‘You’re going to have a rough few days. You’re probably going to have a rough few weeks. If you can get through this, you can get through the rest of the pregnancy.’”*
Theme 4: Focusing on therapeutic rapport, patient readiness, and interest in mental health support	“*Those who just engage with the conversation with me… witness that I’m there just to listen and really validate and they’re like, ‘Oh! Well, she’s cool. She can hear what I have to say!’ They tend to really come back, and it seems like they see me not as a drug treatment counselor but someone who cares about them…I always believe that if I’m able to maintain good rapport, they’ll want to keep coming back… I feel like it’s definitely about the rapport.”*

#### 3.3.1 Theme 1: utilizing the initial clinical encounter

ESS reported that the initial visit with patients includes a comprehensive psychosocial interview, including substance use history. ESS use the initial clinical encounter to intentionally introduce the topic of prenatal cannabis use and develop intervention plans that balance the patients’ needs and goals with the risks of using cannabis during pregnancy. While all ESS assess the same topics, many ESS shared their own individual strategies for intake sessions and introducing sensitive topics. Several ESS observed that assessing alcohol use first is helpful, given its high prevalence, followed by a transition to cannabis use. Some ESS set an agenda at the start of the appointment and review toxicology and/or self-report mental health and substance use screening results. Other ESS introduce mental health and stressors first, and follow up with queries about how patients typically cope with stress. Many ESS emphasized approaching the intake holistically, and learning about the patient’s life outside of cannabis use was critical for developing rapport.

ESS also reported using the initial encounter to assess patients’ goals around cannabis use. Most ESS observed that while many patients are open to completely abstaining during pregnancy and/or had already quit upon confirmation of pregnancy, a few remain reluctant to change their level of use and are uninterested in addressing use with a clinician. Other patients who continue to use cannabis during pregnancy are open to reducing or abstaining, particularly by the third trimester and/or delivery, but desire support. ESS stated that while their general recommendation is to abstain from cannabis use during pregnancy, they work with patients who may not be ready to abstain to determine their intervention goals/plans. Many ESS emphasized the importance of understanding a patient’s motivation to use cannabis and collaborating on intervention goals.

ESS also reported that the initial clinical encounter often includes psychoeducation and interventions to prepare individuals for later in pregnancy and postpartum, as some patients may discontinue ES program participation post-assessment. For example, one ESS described how during her first visit she provides psychoeducation around cannabis use in the third trimester and around the time of delivery, which the patient may not be immediately receptive to but can access in the future: “*So then I talk about maybe the third trimester, maybe thinking about using a little less before delivery, so that the baby can kind of taper down. Because there’s a risk with withdrawal symptoms in the baby. So then I just give some education about that. And, you know if she doesn’t want to do it, then it’s okay, because I’m still giving her the education. And the support. And we’re still skill-building for how to manage things so maybe in the next pregnancy she’s going to remember – because I’ve had that before, too, where they remember what our recommendation is.”*

#### 3.3.2 Theme 2: relying on patient-centered approaches to address prenatal cannabis use

ESS described approaches to address prenatal cannabis use with their pregnant patients directly. Patient-centered approaches—such as MI, harm reduction, and tailored psychoeducation—were among the most common. ESS reported that MI is essential for assessing patients’ readiness and goals for quitting or reducing use during pregnancy. ESS further highlighted affirming and validating patients, emphasizing willingness to change, and exploring patients’ motivations for use, particularly if their motivation was to alleviate pregnancy-related symptoms (such as nausea, sleep problems, or pain), to be able to suggest alternative means of coping.

ESS also described utilizing harm reduction and psychoeducation strategies to align with patients who were not ready to quit using cannabis altogether but were open to reducing use or developing an intervention plan with alternative coping strategies. ESS reported that flexibility and problem-solving were helpful frames for implementing harm reduction and psychoeducation. Several ESS observed how psychoeducation could be an empowering tool for patients by providing them with information to make an informed choice about using cannabis.

ESS offered strategies around delaying use, replacing use with another activity, reducing frequency and quantities of use, and switching modes of use. An ESS described their experience with one patient who reduced their use: “*She went from 10 times a day – she’s been using since she was 12, and she’s 26 – and as soon as she found out she was pregnant, her first pregnancy, she went to two, on her own. And she wants to stop. I said, ‘What’s your goal to stop?’ She said, ‘I want to stop during the first trimester’ So, I said, ‘Keep doing what you’re doing. I want to reinforce these changes you’ve made are awesome. You must be so proud of yourself.’ I don’t want to be her cheerleader. I want to reinforce what she’s done… And I think she was stuck, kind of like, ‘How can I do more harm reduction?’ after she went from 10 to 2. So I said, ‘What if you continue to cut it, like you just did, maybe to once a day or every other day?’ And she goes, ‘I didn’t think about that. Okay, I can do that.'”*

Another ESS shared the impact of a harm reduction mindset: *“I think the thing with harm reduction is just more of a mindset shift that can be helpful for patients. Sometimes patients are like, ‘Well, I’ve already used it through half of my pregnancy, so I’m not going to do any good. It’s not helpful at this point; I’ve already ruined it,’right?…' I like to use the phrase that ‘Every single day that baby is not exposed to THC, you are decreasing the risk of them having any negative side effects from the THC exposure. Every single day that they are going without that exposure is helping them develop in a healthier way.’”*

#### 3.3.3 Theme 3: leveraging expertise in complementary areas

Prior to serving as an ESS, clinicians had diverse clinical experiences in sleep hygiene, family/child therapy, child development, child protection services, and trauma. ESS reported leveraging their expertise in complementary areas to support care (e.g., sleep, family/child therapy, child development, trauma) to help patients gain skills to address their pregnancy symptoms in more adaptive ways rather than using cannabis. ESS report delivering interventions that address pregnancy symptoms in lieu of cannabis use, which patients are often unaware of, but are open to trying. Diverse expertise provides unique opportunities to complement and support ESS’ primary role of addressing prenatal substance use.

Importantly, complementary training and expertise are also particularly helpful when patients report using cannabis to address pregnancy-related symptoms. For example, ESS reported that many patients continue using cannabis to alleviate pregnancy-related symptoms such as nausea, pain, and sleep problems. At the same time, ESS reported many patients do not fully explore alternatives to using cannabis to address these symptoms. ESS described offering interventions to replace cannabis use, including digital applications/health tools, referrals to medical providers, lifestyle changes (e.g., diet, exercise), sleep hygiene, and mindfulness. ESS observed that once they initiate this conversation, many patients are open to considering alternatives to cannabis use. ESS reported that other patients might have previously considered alternatives to using cannabis to address pregnancy symptoms but struggled to implement these changes. Such patients appreciate the support and accountability of working with a clinician to implement these strategies. One ESS offered the value of stress management strategies as a future parent: *“I will talk with them about stress management as a family value; that they are embarking on a journey to be the primary guardian of a little human being who is going to [have] stress in their life. And their kid isn’t going to do what they say. Their kid is going to do what they do. So if they can start, as a family value, stress management one minute before dinner or one minute before bed. The whole family does it. It’s a family value.”*

#### 3.3.4 Theme 4: focusing on psychotherapeutic factors (therapeutic rapport, patient readiness, and interest in mental health support)

ESS identified psychotherapeutic factors for engaging patients in care, including therapeutic rapport, patient readiness, and interest in mental health support. ESS reported that because prenatal cannabis use is stigmatized, many patients may be reluctant to participate in prenatal substance use interventions. The focus on rapport and care for the patient’s overall well-being facilitated more meaningful discussion. It allowed ESS to transition adeptly into more sensitive topics such as prenatal cannabis use.

Besides conducting a comprehensive assessment, ESS prioritize developing therapeutic rapport quickly. ESS also acknowledged the value of timing in a patient’s readiness to participate and leveraging pregnancy as a unique and critical opportunity to change their cannabis use, particularly for individuals who were ambivalent or had made efforts to quit use in the past. One ESS noted pregnancy as a unique motivator for reducing cannabis use: *“Just using this very unique time in their life where this may be the only time that they’ve even considered not using cannabis, and their willingness and readiness to try other things that they maybe weren't ever going to try.”* Other ESS described how some patients were simply not ready or interested in changing their cannabis use and/or participating. Many ESS noted that a patient’s openness to talk about prenatal cannabis use and readiness to change during the initial visit related to the likelihood of follow-up appointments. Some ESS noted that navigating information about cannabis use risks is related to a patient’s openness. For example, ESS found that blanket statements about cannabis harms were not always compelling to patients, particularly for those who had done their own research or knew others with personal experiences using cannabis during pregnancy. ESS described being intentional around sharing psychoeducation and acknowledging established evidence about risks and limitations of current research.

ESS noted the value of the ES program as potentially a patient’s only mental health support during pregnancy, often evidenced by the patient’s responsiveness when asked about mental health generally. One ESS described supporting patients’ wellness: “*At the end I say, ‘I’d really like to meet with you again, if you’re willing.’ And I really try to focus that when we meet again 'we’re going to be talking about other things; about how you’re doing in your pregnancy. It’s not all just going to be focused on this [cannabis use]. I certainly will ask you about it. We will talk about it. But you know, I try to look at it as a big picture of support. It’s not just support with the cannabis.'”*

## 4 Discussion

Through a mixed-methods approach, we explored the perspective of mental health clinicians who work with patients who use cannabis during pregnancy. Survey results demonstrated that ESS perceive patients’ diverse motivations for use and rely primarily on peers for information. Qualitative themes highlighted that ESS leverage expertise in complementary areas and intentionally plan initial clinical encounters to introduce, assess, and intervene around prenatal cannabis use. ESS also emphasize that psychotherapeutic factors (therapeutic rapport, patient readiness, and mental health) contribute to patient engagement. Both qualitative and survey results indicated that ESS find patient-centered interventions such as MI, harm reduction, and psychoeducation to be the most helpful in addressing cannabis use with patients.

For therapeutic factors, such as rapport and patient readiness, the perspectives of ESS clinicians are consistent with current literature on psychotherapy factors in substance use treatment (Flora, 2013, Meier et al., 2005) and complement the experiences of patients who use cannabis during pregnancy. Focus groups with pregnant patients engaging in cannabis use demonstrate that the clinician approach, including rapport and a nonjudgmental stance, plays a role in patients willingness to discuss their use with their clinician (Foti et al., 2023). Our study found that clinicians use patients’ motivations and needs to better understand the context of their cannabis use, which felt more personal and helpful compared to a more general recommendation. This strategy appeared consistent with both rapport-building and acknowledging patients’ anecdotal experiences with cannabis use. Other settings might consider prioritizing rapport-building alongside standardized assessments and psychoeducation. Many physicians are likely aware of interventions such as MI and harm reduction to explore a patient’s readiness to not use cannabis (Groff et al., 2023). Training in and incorporating MI and harm reduction strategies throughout prenatal care and across disciplines might further facilitate behavior change. Relatedly, clinicians highlighted how harm reduction interventions complemented the ES program’s patient-centered approach. Pregnancy offered a unique opportunity to change cannabis use. ESS worked with patients to consider harm reduction strategies they had never considered before, such as delaying use or replacing use with other activities. Other clinicians might leverage and highlight pregnancy as an opportunity for patients to change their cannabis use, particularly for those who may not have considered making a change previously.

Prior qualitative work with pregnant patients also indicates patients’ motives to self-medicate with cannabis to relieve pregnancy symptoms, particularly nausea, appetite, and stress (Chang et al., 2019, Foti et al., 2023); other quantitative studies show that pregnant patients with nausea and vomiting, depression and anxiety, have elevated rates of prenatal cannabis use (Young-Wolff et al., 2018, Young-Wolff et al., 2020). Our results similarly found ESS recognize patients’ diverse motives for use and are intentional about introducing strategies (e.g., psychoeducation, wellness, sleep hygiene, stress management) that offer patients alternative ways to manage their symptoms without cannabis. The ES program uniquely creates a structure to address prenatal substance use and mental health as a routine part of prenatal care. Other systems may consider how creating similar organizational structures could support clinicians who work with patients using cannabis during pregnancy. Such efforts may also foster a multidisciplinary perspective to treating pregnancy symptoms and facilitating referrals for additional support during pregnancy. Recruiting clinicians with diverse training and providing access to interventions (e.g., sleep, stress management, mental health), in addition to substance use interventions, could engage patients who might be otherwise reluctant to directly address their cannabis use during pregnancy.

Our results are consistent with prior research that suggests pregnant individuals who use cannabis rely on experiences of friends and family to learn about cannabis and desire clear and evidence-based information (Jarlenski et al., 2016, Macario and Thomas, 2022, Taneja et al., 2022). Similarly, ESS reported encounters with patients who turned to peers for information and to navigate potential gaps in knowledge about cannabis’ effects. This finding suggests peer-to-peer support and/or interventions might appeal to many pregnant patients. Additionally, clinicians might consider that patients may weigh peers' opinions over clinical recommendations. Clinicians may not always discuss cannabis use with pregnant patients, even when patients disclose use, focus on the legal consequences instead of the health effects (Holland et al., 2016, Woodruff et al., 2021). Further research is warranted to examine how clinicians may be perceived as more accessible and trusted resources for health information about cannabis use during pregnancy, bolstered with patient-centered interventions.

The present findings must be considered in light of study limitations. The sample was limited to a small group of mental health clinicians working in a specialized prenatal substance use screening and intervention program embedded within prenatal care at KPNC. The perspectives of these clinicians may not generalize to those who work with pregnant patients outside of KPNC or in other settings (e.g., specialty addiction treatment). Importantly, while KPNC encompasses a diverse patient population, the sample of clinicians in our study was primarily female, White, and middle-aged. This has important implications for how clinicians may be navigating conversations around cannabis use, and the willingness of patients to engage openly with a provider. Furthermore, results may be limited in generalizability to providers of other backgrounds. Nevertheless, study participants had extensive expertise working with patients who use cannabis during pregnancy and offered critical clinical insights. Study participants were also providing clinical services within a state that has legalized cannabis use. Experiences of clinicians who work with pregnant patients in states with different cannabis policies may differ, particularly those with stricter reporting guidelines for Child Protective Services (CPS) than California. Even in states where cannabis is legal, concerns about CPS reporting in the context of prenatal cannabis use remain, particularly for low-income and minoritized individuals (Ceasar et al., 2023). Yet, focus groups on cannabis legalization do demonstrate that patients are more open to having conversations about their prenatal cannabis use with their clinicians after legalization (Young-Wolff et al., 2022).

## 5 Conclusion

This mixed-methods study explored the perspectives of mental health clinicians working with pregnant patients who used cannabis. Survey and qualitative results indicated that patient-centered interventions, therapeutic factors, and understanding patients’ motives to use cannabis are critical areas for engaging patients in prenatal substance use interventions. Opportunities for patient engagement and intervention include understanding patients’ motives for use, structuring and prioritizing initial clinical encounters, enhancing patient readiness, offering alternatives to cannabis use, and bolstering clinical information for patients. The present study provides expert insights that can benefit clinicians across all settings who seek to better engage pregnant patients who use cannabis.

## CRediT authorship contribution statement

**Altschuler Andrea:** Writing – review & editing, Supervision, Methodology, Formal analysis, Data curation. **Does Monique B.:** Writing – review & editing, Project administration, Methodology, Funding acquisition, Formal analysis, Conceptualization. **Mian Maha N:** Writing – review & editing, Writing – original draft, Methodology, Investigation, Funding acquisition, Formal analysis, Data curation, Conceptualization. **Young-Wolff Kelly C.:** Writing – review & editing, Supervision, Investigation, Formal analysis, Conceptualization. **Satre Derek D.:** Writing – review & editing, Validation, Supervision. **Asyyed Asma H.:** Writing – review & editing, Validation, Supervision. **Castellanos Carley:** Writing – review & editing, Resources, Methodology. **Ansley Deborah R.:** Writing – review & editing, Resources, Methodology, Conceptualization. **Green Andrea:** Writing – review & editing, Validation, Resources, Methodology, Conceptualization.

## Funding

This study was supported by the Kaiser Permanente Northern California Division of Research 2023 Behavioral Health and Infectious Disease Section Pilot Grant and the 10.13039/100000026National Institute on Drug Abuse, Award Number T32DA007250.

## Declaration of Competing Interest

Authors have no conflicts of interest to disclose.
